# Deep learning based digital pathology for predicting treatment response to first-line PD-1 blockade in advanced gastric cancer

**DOI:** 10.1186/s12967-024-05262-z

**Published:** 2024-05-08

**Authors:** Yifan Liu, Wei Chen, Ruiwen Ruan, Zhimei Zhang, Zhixiong Wang, Tianpei Guan, Qi Lin, Wei Tang, Jun Deng, Zhao Wang, Guanghua Li

**Affiliations:** 1 Department of Gastrointestinal Surgery, First Affiliated Hospital of Sun Yat-sen University, Zhongshan 2nd Street, No. 58, Guangzhou, 510080, 86 Guangdong China; 2 Department of Oncology, First Affiliated Hospital of Nanchang University, Nanchang, 330006 Jiangxi Province China; 3 Department of Pathology, First Affiliated Hospital of Sun Yat-sen University, Guangzhou, Guangdong China; 4 Guangdong Provincial Key Laboratory of Digestive Cancer Research, Digestive Diseases Center, Scientific Research Center, The Seventh Affiliated Hospital of Sun Yat-sen University, Shenzhen, Guangdong China; 5 Department of Gastrointestinal Surgery, Affiliated Cancer Hospital & Institute of Guangzhou Medical University, Guangzhou, Guangdong China

**Keywords:** Digital histopathological images, AI, Deep learning, Advanced gastric cancer, Immune Checkpoint inhibitors, ICIs, Chemotherap, PD-1

## Abstract

**Background:**

Advanced unresectable gastric cancer (GC) patients were previously treated with chemotherapy alone as the first-line therapy. However, with the Food and Drug Administration’s (FDA) 2022 approval of programmed cell death protein 1 (PD-1) inhibitor combined with chemotherapy as the first-li ne treatment for advanced unresectable GC, patients have significantly benefited. However, the significant costs and potential adverse effects necessitate precise patient selection. In recent years, the advent of deep learning (DL) has revolutionized the medical field, particularly in predicting tumor treatment responses. Our study utilizes DL to analyze pathological images, aiming to predict first-line PD-1 combined chemotherapy response for advanced-stage GC.

**Methods:**

In this multicenter retrospective analysis, Hematoxylin and Eosin (H&E)-stained slides were collected from advanced GC patients across four medical centers. Treatment response was evaluated according to iRECIST 1.1 criteria after a comprehensive first-line PD-1 immunotherapy combined with chemotherapy. Three DL models were employed in an ensemble approach to create the immune checkpoint inhibitors Response Score (ICIsRS) as a novel histopathological biomarker derived from Whole Slide Images (WSIs).

**Results:**

Analyzing 148,181 patches from 313 WSIs of 264 advanced GC patients, the ensemble model exhibited superior predictive accuracy, leading to the creation of ICIsNet. The model demonstrated robust performance across four testing datasets, achieving AUC values of 0.92, 0.95, 0.96, and 1 respectively. The boxplot, constructed from the ICIsRS, reveals statistically significant disparities between the well response and poor response (all p-values < = 0.001).

**Conclusion:**

ICIsRS, a DL-derived biomarker from WSIs, effectively predicts advanced GC patients’ responses to PD-1 combined chemotherapy, offering a novel approach for personalized treatment planning and allowing for more individualized and potentially effective treatment strategies based on a patient’s unique response situations.

**Supplementary Information:**

The online version contains supplementary material available at 10.1186/s12967-024-05262-z.

## Introduction

Gastric cancer (GC) is one of the most common malignancies of the digestive system. Globally, it accounts for approximately 1.08 million new cases annually, ranking fifth in cancer incidence. Moreover, GC ranks third in cancer-related mortality, causing over 760,000 deaths each year [1]. Due to its nonspecific symptoms, 80–90% of GC patients are diagnosed at an advanced stage during their initial presentation [2], resulting in poor prognoses [3, 4]. Surgery is the primary treatment method for GC. However, the 5-year survival rate for postoperative advanced GC patients is approximately 60–70% in East Asian countries [5, 6], whereas in Western countries, it remains at only 20-30% [7, 8].

In the past, systemic chemotherapy serves as the cornerstone of treatment for advanced GC, yielding a median overall survival of approximately 12 months among patients subjected to conventional chemotherapy [9]. Consequently, there is an urgent need for more effective treatment strategies to improve the survival outcomes of advanced unresectable GC patients. In recent years, the emergence of targeted therapies and immunotherapeutic approaches holds promise for improving this status quo.

The immune system effectively eliminates most invading pathogens and toxic substances within the body while maintaining self-tolerance to normal tissues. Self-tolerance is achieved through the immunosuppressive effects of immune checkpoint pathways. Among the regulatory mechanisms governing immunosuppression, the programmed cell death protein 1 (PD-1) pathway, first discovered by Ishida et al. in 1992 [10], stands out as one of the most extensively studied pathways till today. Over the past three decades, numerous studies have highlighted the critical role of PD-1 in negative immune regulation and the maintenance of peripheral self-tolerance [11–14]. However, it has been discovered that the development of tumors is closely related to the immune system. In 2002, Iwai et al. provided the initial evidence of PD-1 signaling pathway involvement in mediating tumor immunity [15], and it is now widely recognized that tumor cells can also exploit immune checkpoints to suppress tumor immunity and evade immune surveillance [16, 17].

Today, PD1 inhibitors have become a common component of cancer first-line treatment options for various malignancies [18]. In 2021, a prospective clinical trial, CheckMate 649, which enrolled a total of 2,687 patients, confirmed that the combination of Nivolumab with chemotherapy significantly improved the prognosis of previously untreated, unresectable, non-HER2-positive gastric adenocarcinoma patients [19]. In 2022, the National Comprehensive Cancer Network (NCCN) clinical practice guidelines for gastric cancer included the use of anti-PD-1 (Nivolumab) agents in combination with fluoropyrimidine and platinum-based drugs as one of the first-line treatment options for systemic treatment of metastatic or locally advanced gastric cancer (in the absence of local treatment indications) [20]. This highlights the important role of immunotherapy in the management of advanced GC. However, not all patients receiving immunotherapy exhibit favorable treatment responses, and the limitations imposed by unnecessary treatment side effects and tumor progression during ineffective treatment hinder the further application of anti-PD-1 therapy [21]. Thus, effective predictive tools and indicators are needed in clinical practice to characterize and select advanced GC patients who are sensitive to PD-1 inhibitor plus chemotherapy, aiming to improve the efficiency of immunotherapy in clinical applications.

The emergence of Whole Slide images (WSIs) has brought about a paradigm shift in the field of digital pathology. These digitized WSIs have enabled the systematic extraction of histochemical and immunohistochemical data from tissue specimens [22]. Through the utilization of artificial intelligence (AI), morphological characteristics can be converted into digital data that are well-suited for machine learning [23]. More specifically, Deep learning(DL), a subset of machine learning, has assumed an significant role in the analysis of pathological images [24]. Convolutional neural networks (CNNs), which represent a DL algorithm, possess the capability to discern subtle visual intricacies and extract fundamental features critical for expert-level comprehension. Their application to the field of pathology has yielded outstanding outcomes, particularly in the domains of cancer detection, lesion classification, and prognostic forecasting [25–28].

In this retrospective study, we collected WSIs of Hematoxylin and Eosin (H&E)-stained biopsy samples from patients diagnosed with advanced GC from multiple medical centers. The objective was to establish a classifier using DL methods and theories to predict the efficacy of immunotherapy in patients with advanced GC receiving first-line PD-1 inhibitors combined chemotherapy. To the best of our knowledge, this study represents the first research endeavor aimed at predicting patient sensitivity to first-line PD-1 inhibitors combined chemotherapy based on pre-treatment biopsy samples.

## Methods

### Patients cohort and ethics approval

Patients with advanced GC were collected from four large medical centers, namely, the First Affiliated Hospital of Sun Yat-sen University (FAH-SYSU), the First Affiliated Hospital of Nanchang University (FAH-NCU), the Seventh Affiliated Hospital of Sun Yat-sen University (SAH-SYSU), and the Affiliated Cancer Hospital of Guangzhou Medical University (ACH-GZMU). The inclusion and exclusion criteria for the study cohort were as follows: (1) A confirmed pathological diagnosis of gastric adenocarcinoma. (2) Locally advanced, recurrent, or metastatic disease that cannot be resected, with no surgical indications at the time of diagnosis and no prior surgical treatment [20]. (3) HER2 expresses negative. (4) Receipt of at least three whole cycle of first-line PD-1 inhibitors combination chemotherapy in a continuous manner (approximately three to four months), with no prior exposure to alternative treatment before initiating immunotherapy. (5) Pre-treatment endoscopic biopsy performed, with available HE-stained pathological slides as specimens. (6) Presence of evaluable lesions, with at least baseline-enhanced Computed Tomography (CT) scans prior to treatment initiation and follow-up enhanced CT scans after treatment cycles. All patients who did not meet the criteria were excluded from the study. According to the criteria, a total of 139, 90, 25, 10 patients were enrolled from FAH-SYSU, FAH-NCU, SAH-SYSU, and ACH-GZMU, respectively, during the period spanning from March 2021 to January 2024. PD-1 inhibitors used conclude Nivolumab, Camrelizumab, Toripalimab, Pembrolizumab, and Stintilimab.

The evaluation of the efficacy of PD-inhibitors combined chemotherapy was performed according to iRECIST 1.1 criteria [29] using baseline-enhanced CT and follow-up CT scans. When calculating lesion changes, all assessable lesions were included, encompassing primary lesions, assessable lymph node metastases, and distant metastases (if present). Patient treatment responses were categorized as immune Complete Response (iCR), immune Partial Response (iPR), immune Stable Disease (iSD), and immune Progressive Disease (iPD). Given that our primary objective was to identify the patient population potentially benefiting from this treatment course, we combined iCR and iPR into the category ‘Well Response,’ while iSD and iPD were defined as ‘Poor Response’. It is worth noting that in clinical practice, there are instances where patients exhibit pseudoprogression during immunotherapy [30], meaning that initially, there may be an apparent lack of response or even tumor progression, but subsequent treatment leads to significant regression. Compared to the traditional RECIST 1.1 criteria [31], iRECIST 1.1 incorporates characteristics unique to immunotherapy, providing a more comprehensive evaluation method, especially in the context of pseudo-progression. Therefore, all patients evaluated for iPD were evaluated for at least another 4 weeks according to the iRECIST guidelines. All assessments were conducted by Professors Zhao Wang, Guanghua Li, and Zhixiong Wang, each of whom possesses over 15 years of clinical experience in gastrointestinal surgery.

The study was approved by the Ethics Committee of FAH-SYSU in China and followed the Declaration of Helsinki. Sample collection was authorized by the ethics board of each institution (Ethics Review [2022] No. 090).

### Datasets

A total of 313 formalin-fixed paraffin-embedded pathological slides stained with H&E, obtained from the above-mentioned 264 patients with advanced GC, were included in this research. All these slides acquired from endoscopic biopsies. For the training of DL models, 80% of the FAH-SYSU slides were randomly splited as the training dataset, while the remaining 20% were reserved for internal testing purposes. The WSIs from FAH-NCU, SAH-SYSU and ACH-GZMU served as independent external testing datasets to assess the models’ performance. Prior to formal training, 20% of the training dataset was set aside as a validation dataset to experimentally determine the optimal training hyperparameters, which encompassed learning rate, optimizer, regularization, and batch size. Throughout the formal training process, the validation dataset continued to be an integral part of the training set.

### Sample preparation

Slides were scanned as WSIs using KF-PRO-020 scanner (KONFOONG Biotech, China) and NanoZoomer S210 scanner (Hamamatsu Photonics K.K., Japan). WSIs raw formats include TIF and NDPI. Each WSI was acquired at the highest resolution of 40 x magnification with a corresponding pyramid resolution from the bottom level (40 x) to the top level (1 x). each pixel at 40 x magnification represented a physical size of approximately 0.25 × 0.25 µm^2^. WSIs with 40 x resolution contained on the order of 100,000 × 100,000 pixels, which were multiple orders of magnitude larger than the common pathological images. If possible, constructing a DL model for the entire WSI is the ideal choice. However, given the current computational performance limitations and the requirements of embedded applications, the input resolution for image classification DL models are restricted (e.g., 224, 256, 384 pixels). Therefore, this study employed segmented instances to train the DL models.

A total of 126 slides in the training cohort, 20 in the validation cohort, 33 in the internal testing cohort, and 154 in the external testing cohort were analyzed. When dealing with biopsy specimens, there is a common challenge of distinguishing between normal tissue, compressed/fragmented tumor tissue, non-glandular tissue, and fatty tissue. Moreover, technical issues such as overlap, suboptimal staining, and out-of-focus regions can introduce model biases. To address these challenges, each slide containing GC tissue underwent a meticulous review conducted by an expert pathologist, Zhimei Zhang, who boasts two decades of experience in the field of pathology. Then, target regions were annotated as regions of interest (ROIs) using KF-Viewer and NanoZoomer-Viewer software. These annotations were subsequently reviewed by another expert, Guanghua Li. Then, the position coordinates were saved in XML format and a corresponding TIFF format mask was generated with the same pyramid resolution as the WSI.

We utilized the sliding window approach to extract ROIs from the WSI at 40x magnification. The window width was set to 1024 pixels with a stride of 512 pixels, aiming to obtain the widest possible view and relatively complete tissue structures. Each 1024 × 1024 pixel region at 40x magnification was saved in JPEG format and named as “tile” if the overlap between the mask region and the corresponding WSI region exceeded a threshold of 0.6. At last, the average number of tiles per patient was 473, with a standard deviation of 354. For each tile in the training datasets, the following transformations were applied before inputting it into the model: (1) Resize to 256 × 256 pixels. (2) Convert to tensor. (3) Normalize using the image mean and standard deviation (0.485, 0.456, 0.406; 0.229, 0.224, 0.225). (4) Random horizontal and vertical flip with a probability of 10%. (5) Random rotation by 10 degrees to enhance data diversity. As for the testing datasets, only the first three above-mentioned preprocessing steps were applied, and no data augmentation was performed. This ensures that the test tiles remain consistent and is not artificially altered by data augmentation techniques.

### Base classification models

CNNs have been one of the earliest and most effective DL methods for handling image data [32]. In recent years, Vision Transformer (ViT) models have also demonstrated impressive performance in image classification tasks [33]. In this study, we evaluated two CNN-based models, namely EfficientNet-B4 and DenseNet121 as well as one Transformer-based models, Swin Transformer V2, to evaluate the underlying features present in the image tiles and perform classification based on the outcomes. This diverse set of models allows us to comprehensively assess the features within the pathological images and choose the most suitable model for addressing the task at hand.

Due to the relative scarcity of pathological data, and to achieve better results in a shorter timeframe while preventing overfitting, we opted for a transfer learning method. We loaded pre-trained weights of three different models available in the Timm library (https://github.com/rwightman/pytorch-image-models), which were originally trained on ImageNet (https://image-net.org/). We customized the fully connected layer to include two neurons and applied the Softmax activation function in the output layer. This configuration enables the model to output the probabilities of two classifications, ensuring that the sum of probabilities for both categories equals 1, and the model is fine-tuned according to the complexity of the task. During training, we fine-tuned the model through backpropagation. Each model was trained 100 epochs, utilizing the SGD optimizer with a learning rate of 1e-6. The loss function employed was cross-entropy, and the batch size was set to 32.

The degree of ICIs combined chemotherapy response for each patient is calculated by taking the average of the predicted probabilities of ‘well reaction’ from all tiles within the respective WSI. These values range from 0 to 1, where a value closer to 1 indicates that the patient is more sensitive to PD-1 inhibitor combined chemotherapy and may potentially have a better response, while a value closer to 0 suggests that the patient is less sensitive to this strategy, and the effectiveness of immune therapy combined chemotherapy for these patients may be lower.

### Immune checkpoint inhibitors response network and the ensemble model

In the internal testing dataset, we compared the overall predictive performance of the three models. The probabilities output by each model were averaged to create an ensemble model. This ensemble model integrates the predictions of these three models, aiming to maximize the balance of predictive biases, enhance generalization performance, and improve accuracy. After evaluation, the ensemble model demonstrated the most outstanding and well-balanced predictive performance. When all tiles from each patient are input to the ensemble model, the resulting averaged prediction probabilities are computed to generate the prediction score, referred to as the Immune Checkpoint Inhibitors Response Score (ICIsRS). Therefore, this model was also named as ICIsNet. ICIsRS represents a continuous quantified value. We plotted ROC curves on the internal test dataset on tile level and WSI level, furthermore, we determined the optimal cutoff value based on the ROC curve of WSI-level. If ICIsRS exceeds the cutoff value, the prediction indicates a patient’s sensitivity to first-line PD-1 inhibitor combined chemotherapy. Conversely, if ICIsRS is less than or equal to the cutoff threshold, it suggests that the model predicts the patient as insensitive to them. T-tests was employed to examine the differences in ICIsRS among four test sets.

### Visualization of ICIsRP -related features

**Fig. 1:**
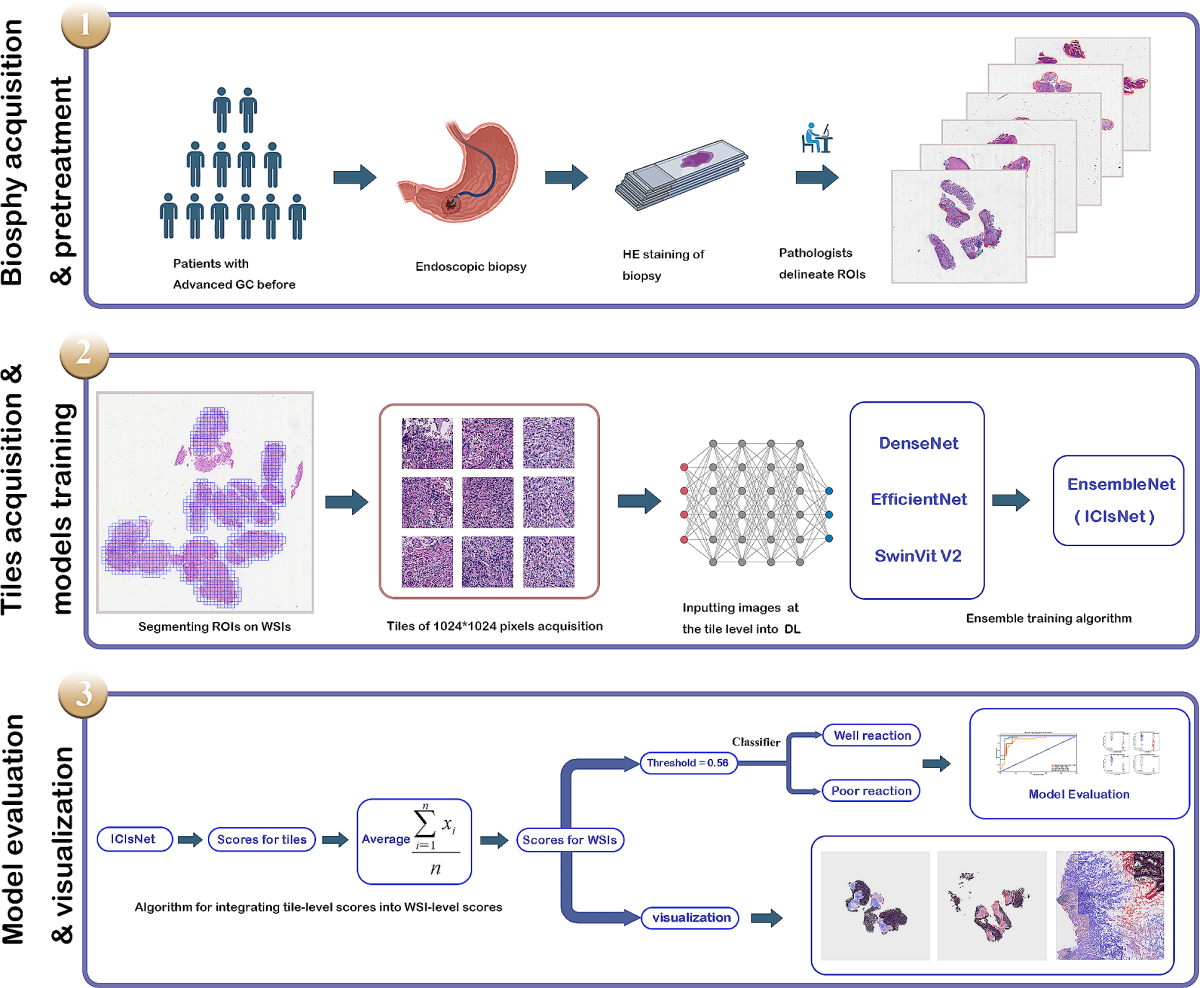
The flowchart and methodology of this research

ICIsNet generates predictive probabilities for each WSIs, and the coordinates of each tile within the WSI were stored in their respective JPEG file names. Employing the OpenCV (https://opencv.org/**)** and Matplotlib library (https://matplotlib.org/), probability of each tile is represented as a color block. A color spectrum called ‘Coolwarm’ ranging from blue to red represents the response probabilities from low to high. By reassembling these tiles based on their coordinates, we generate a density heatmap of the patient’s response to the WSI, providing us with a visual representation. The detailed methodology is depicted in Fig. 1.

### Statistics

To evaluate the binary classification performance of tile-based models on the test dataset, we employed several key methodologies: 1. Receiver Operating Characteristic (ROC) Curve. 2.Area Under the ROC Curve (AUC). 3.Confusion Matrix to compute essential performance metrics, including accuracy (ACC), sensitivity (SENS), specificity (SPEC), positive predictive value (PPV), and negative predictive value (NPV). 4.F1 Score. 5.Accuracy metric to evaluate the model’s performance. To evaluate the performance of ICIsNet in the binary classification task of predicting patient immunotherapy sensitivity within the test cohort, we applied the same set of metrics. To determine the optimal cutoff value, we generated a Threshold-TPR-FPR curve. Additionally, we created box plots based on the ICIsNet output probability values to provide visual representations of the classification performance.

The models’ construction, training, validation, and visualization were all conducted on a server equipped with two RTX 3090 GPUs and 15 vCPU Intel(R) Xeon(R) Platinum 8358P CPUs @ 2.60 GHz. The server environment ran on Python 3.8 (Ubuntu 18.04) and utilized PyTorch 1.8.1 with CUDA 12.0 architecture for GPU acceleration.

## Result

### Characteristics of patients

Based on the inclusion and exclusion criteria, a total of 264 patients with advanced GC were included in this study. All patients were pathologically diagnosed with advanced GC and did not have indications for surgery. According to the iRECIST 1.1 criteria, among them, 128 patients were categorized into the “well reaction” group, while 136 patients were categorized into the “poor reaction” group. FAH-SYSU, FAH-NCU, SAH-SYSU, and ACH-GZMU each contributed 139, 90, 25, 10 patients, respectively (Table 1). Finally, we obtained a total of 313 WSIs, all these slides were retrieved from endoscopic biopsy (more patients’ characteristics and details were listed in Table 1).

**Table 1:** Characteristics and baseline of patients

	FAH-SYSU	FAH-NAU	SAH-SYSU	ACH-GZMU	Total
Characteristics	Patients (139)	Patients(90)	Patients (25)	Patients (10)	264
**Age**					
**Mean (SD)**	56.7 (12.9)	58.0 (11.7)	59.1 (11.7)	57.0 (12.0)	57.3 (12.9)
**Median [Min, Max]**	58.0 [26.0, 88.0]	58.5 [22.0, 86.0]	59.0 [22.0, 86.0]	59.5 [30.0, 70.0]	58.5 [22.0, 88.0]
**Sex**					
**Female**	35 (25.2%)	35 (38.9%)	24 (39.3%)	1 (10.0%)	77 (29.2%)
**Male**	104 (74.8%)	55 (61.1%)	37 (60.7%)	9 (90.0%)	187 (70.8%)
**Disease region**					
**Localized**	46 (33.1%)	3 (3.3%)	2 (3.3%)	1 (10.0%)	56 (21.2%)
**Metastatic**	93 (66.9%)	87 (96.7%)	59 (96.7%)	9 (90.0%)	208 (78.8%)
**Differentiation**					
**Moderate**	27 (19.4%)	32 (35.6%)	23 (37.7%)	1 (10.0%)	64 (24.6%)
**Poor**	112 (80.6%)	58 (64.4%)	38 (62.3%)	9 (90.0%)	199 (75.4%)
**Primary Site**					
**Lower**	45 (32.4%)	34 (37.8%)	23 (37.7%)	4 (40.0%)	92 (34.8%)
**Middle**	52 (37.4%)	42 (46.7%)	34 (55.7%)	3 (30.0%)	107 (40.5%)
**Upper**	42 (30.2%)	14 (15.6%)	4 (6.6%)	3 (30.0%)	65 (24.6%)
**Reaction**					
**Well reaction**					128 (48.4%)
**iCR**	1 (0.8%)	0 (0.0%)	0 (0.0%)	0 (0.0%)	1 (0.4%)
**iPR**	67 (48.2%)	43 (47.8%)	13 (52%)	4 (40.0%)	127 (48.1%)
**Poor reaction**					136 (51.6%)
**iSD**	38 (27.3%)	29 (32.2%)	4 (16%)	5 (50.0%)	76 (28.8%)
**iPD**	33 (23.7%)	18 (20.0%)	8 (32%)	1 (10.0%)	60 (22.7%)
**WSIs**	159 (50.8%)	100 (31.9%)	44 (14.1%)	10 (3.2%)	313

iCR: immune complete response; PR: immune partial response; iSD: immune stable disease; iPD: immune progressive disease; WSI: whole slide image

### Performance of DL models

**Fig. 2:**
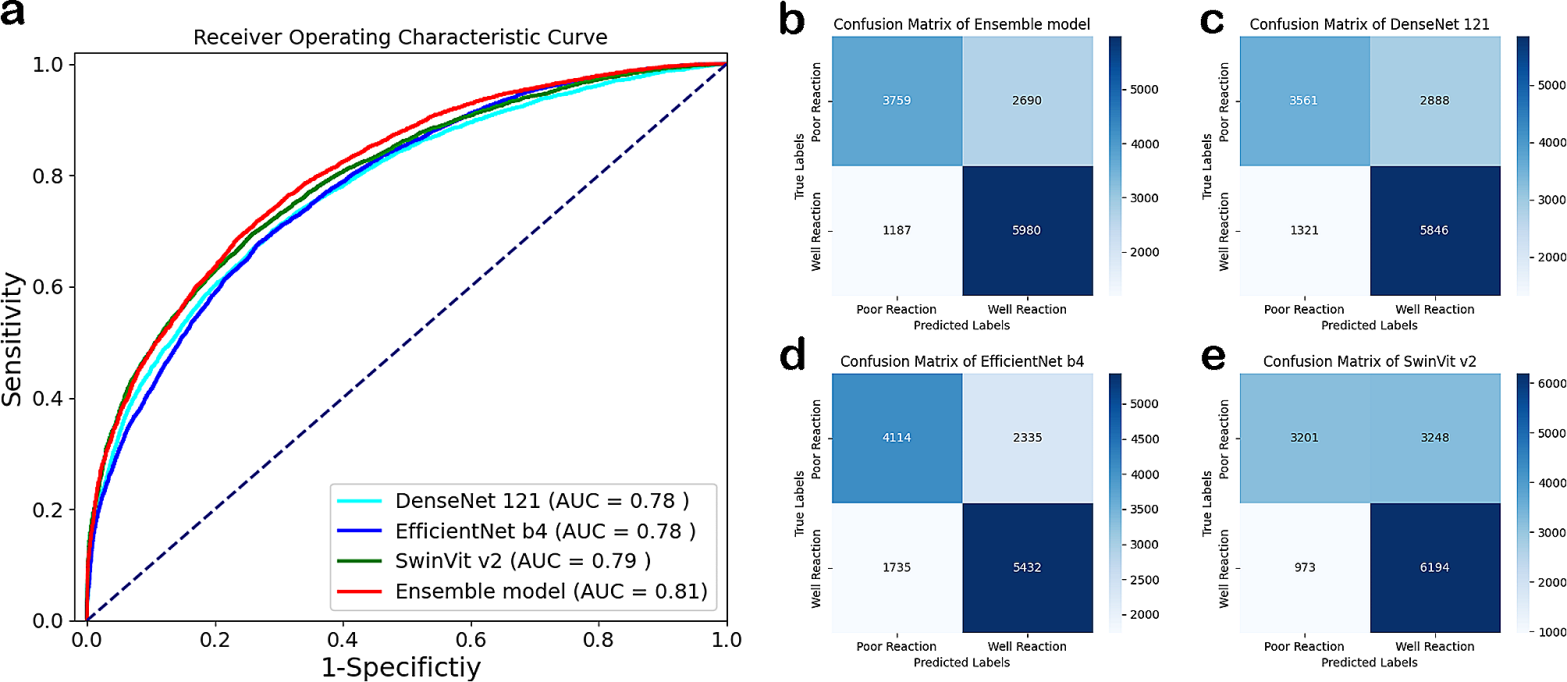
**a**. Receiver Operative Characteristic curves of models for patch-level prediction; **b**. Confusion matrix for the ensemble model in the internal test dataset; **c**. Confusion matrix for the DenseNet121 in the internal test dataset; **d**. Confusion matrix for the EfficientNet b4 in the internal test dataset; e. Confusion matrix for the SwinVit V2 in the internal test dataset

WSIs were segmented into tiles, forming the foundational elements for DL. In our research, we trained the DenseNet121, EfficientNet-B4, Swin-Transformer V2 (tiny) models independently for 100 epochs on 70,016 tiles and evaluated them during testing phases. Furthermore, an ensemble model basing on the three models were constructed and tested as mentioned above. For the internal test cohorts, we calculated the ACC, SENS, SPEC, PPV, NPV, recall and F1 score of each model. In the end, the Ensemble model outperformed in most of these indexes compared with the other three standalone models (with an ACC of 0.715, AUC of 0.805, PPV of 0.689, NPV of 0.760, SENS of 0.834, SPEC of 0.583, and F1 score of 0.755. Figure 2a; Table 2). Furthermore, the confusion matrixes show that the ensemble model has the best comprehensive resolution for different responses (Fig. 2b-e). As a result, we adopted the Ensemble model for subsequent evaluations.

**Table 2:** Comparison of Models’ metrics and selection on the internal test dataset

	ACC	AUC	PPV	NPV	SENS	SPEC	F1 score
**DenseNet121**	0.691	0.776	0.669	0.729	0.816	0.552	0.735
**EfficientNet-B4**	0.701	0.778	0.699	0.703	0.758	0.638	0.727
**Swin Transformer-V2**	0.690	0.795	0.656	0.767	0.864	0.496	0.746
**Ensemble Model**	0.715	0.805	0.689	0.760	0.834	0.583	0.755

ACC: accuracy; AUC: area under the curve; PPV: positive predictive value; NPV: negative prediction value; SENS: sensitivity; SPEC: specificity

**Fig. 3:**
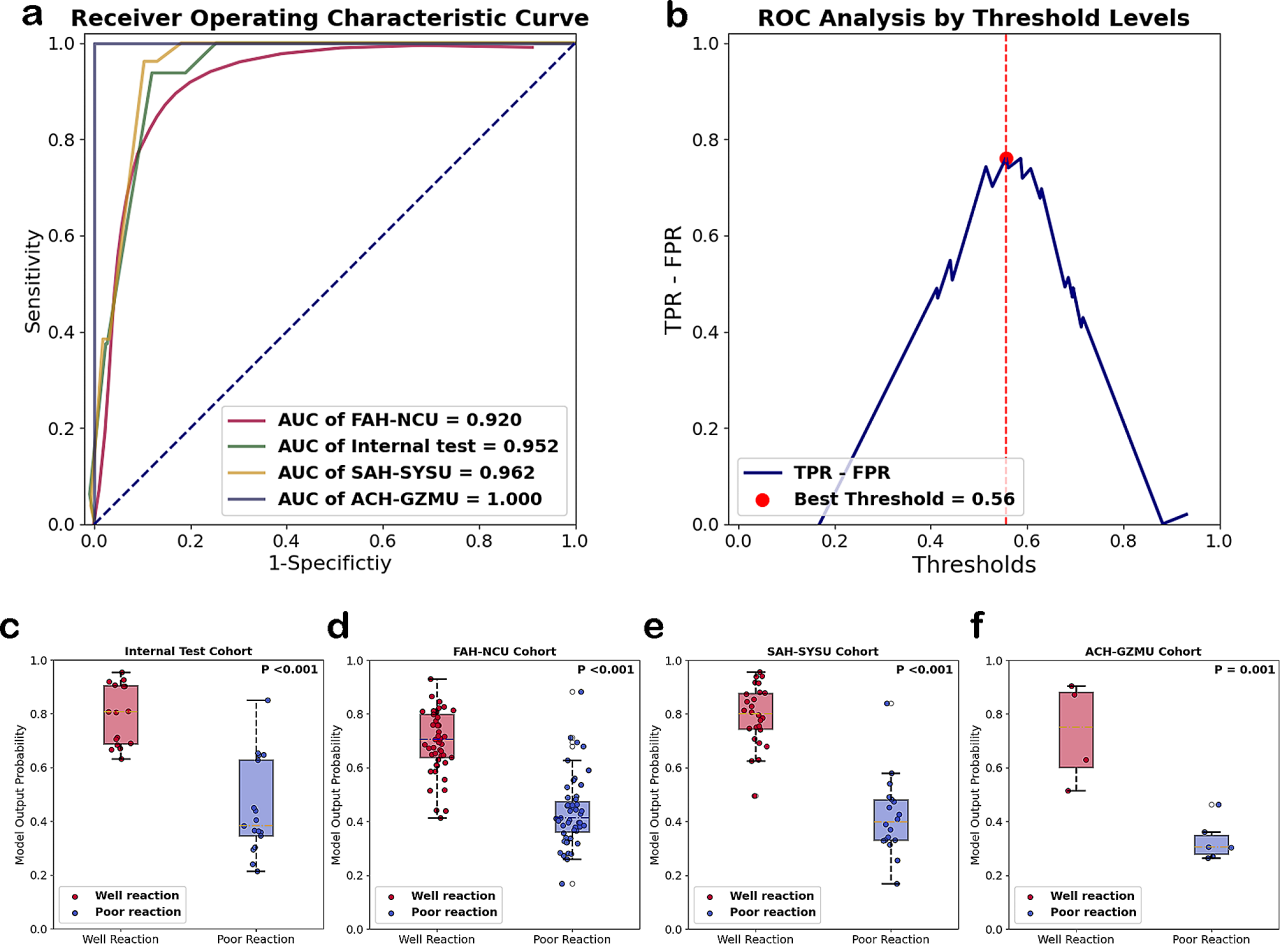
**a**. Receiver Operative Characteristic curves of models for WSI-level prediction among four independent test cohorts; **b**. True Positive Rate - False Positive Rate curve in internal test cohort; **c**. comparison of the ICIsNet output between well and poor responses in the internal test cohort; **d**. comparison of the ICIsNet output between well and poor responses in the FAH-NCU cohort; **e**. comparison of the ICIsNet output between well and poor responses in the SAH-SYSU cohort; f.comparison of the ICIsNet output between well and poor responses in the ACH-GZMU cohort

Basing the Ensemble model, we constructed the ICIsNet to predict the response at the WSI level. After testing, robust AUC scores of 0.952 (internal test dataset), 0.920 (FAH-NAU), 0.962 (SAH-SYSU), and 1 (ACH-GZMU) were achieved for WSI-level predictions, showing the model’s high discriminatory power. The AUC is a crucial measure of a model’s ability to distinguish between classes—in this case, responders and non-responders to first-line PD-1 inhibitors combined with chemotherapy. High AUC values close to 1 indicate excellent model accuracy, as demonstrated by our three external validation cohorts from South China (as shown in Fig. 3a). A comprehensive summary of these results can be found in Table 3. The optimal threshold for ICIsNet predictions was determined on the internal test dataset by maximizing the difference between true positive rate and false positive rate (TPR-FPR), which was found to be 0.56 (Fig. 2b). ICIsRS prediction exceeding this threshold outputted from the ICIsNet to categorize the patient as ‘well reaction’; otherwise, the patient was considered as ‘poor reaction’.

**Table 3:** Evaluation Metrics of ICIsNet in internal test cohort and external test cohort

	ACC	AUC	PPV	NPV	SENS	SPEC	F1 score
**Internal test cohort**	0.848	0.952	0.762	1.000	1.000	0.706	0.865
**FAH-NCU**	0.870	0.920	0.860	0.880	0.876	0.863	0.867
**SAH-SYSU**	0.932	0.962	0.926	0.941	0.962	0.889	0.943
**ACH-GZMU**	0.900	1.000	1.000	0.857	0.750	1.000	0.857

ACC: accuracy; AUC: area under the curve; PPV: positive predictive value; NPV: negative prediction value; SENS: sensitivity; SPEC: specificity

Further investigation into the ICIsRS of each patient in different test sets was conducted. We computed ICIsRS for each WSI in four test cohorts and generated box plots based on these scores. We used t-tests to assess the classification performance. The results, as shown in Fig. 3c-f, indicated significant differences for each test dataset (internal test, FAH-NCU, SAH-SYSU, all with p-values < = 0.001).

**Fig. 4:**
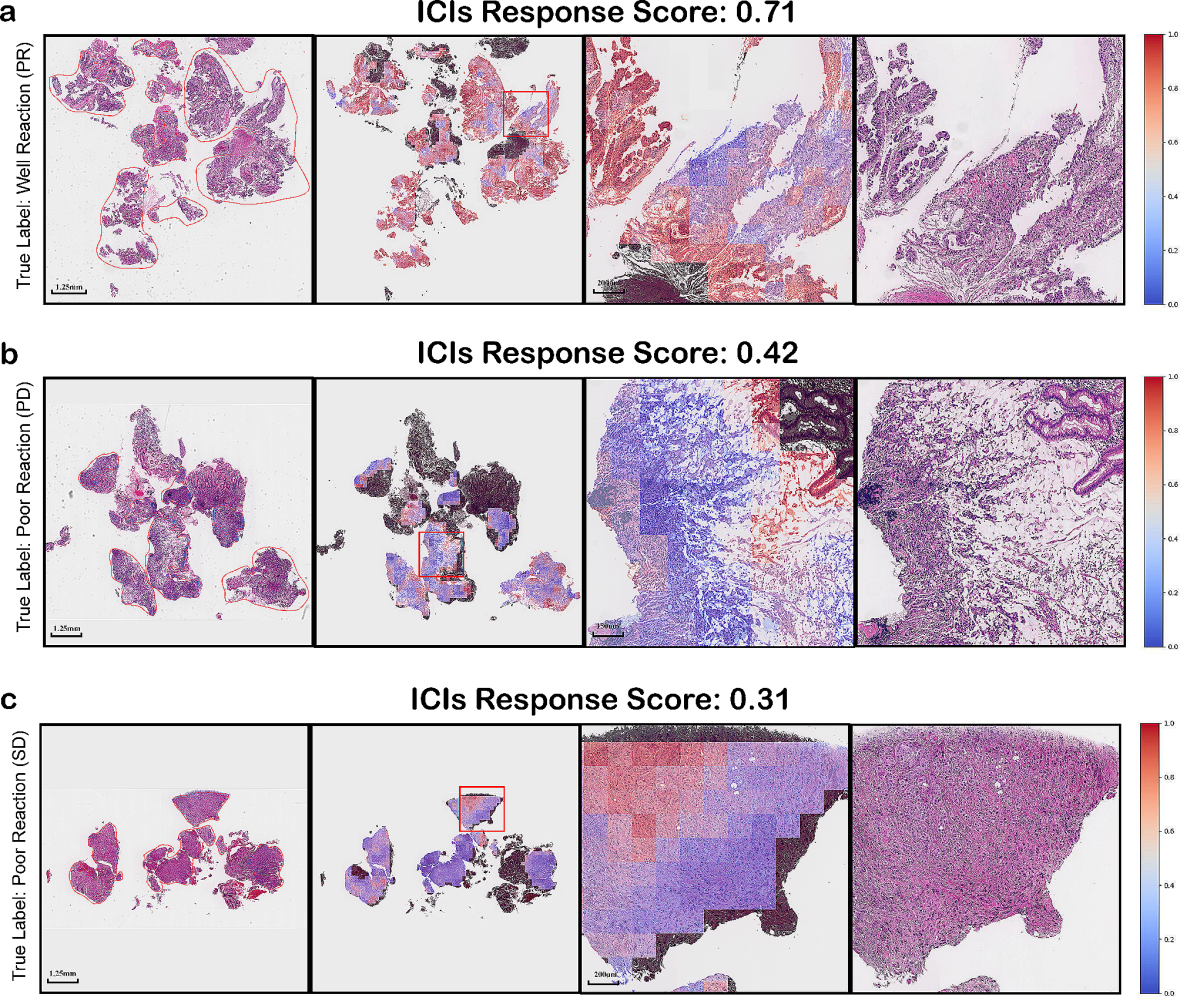
Heat maps and visualization of the incidence of WSIs, from left to right are the original WSIs, the heat map of concerning WSIs, the heat map of the details of WSI, and the details of concerning original WSI. The presence of signet-ring cells or areas of Mucinous cell can be clearly observed leading to a significant weak response to immunotherapy. **a**. an incidence of PR, ICIsRS was 0.71; **b**, an incidence of PD, ICIsRS was 0.42; **c**. an incidence of SD, ICIsRS was 0.31

ICIsNet assigns scores to each WSI based on intricated image features associated with the response of ICIs combined with chemotherapy. Due to the inherently opaque nature of DL models, the emphasized image features by are not explicitly clear. However, when heatmaps of each tile of a whole WSI are superimposed, a more macroscopic observation is obtained. It becomes evident that poorer differentiation or more diffuse tumor tissue characteristics, such as signet ring cells or cells floating in mucin, along with reduced lymphocytic infiltration, are associated with weaker responses to immunotherapy. Conversely, tumor cells that more closely resemble the original normal tissue, coupled with increased lymphocytic infiltration, are likely to elicit better immunotherapeutic responses (Fig. 4).

## Discussion

To the best of our knowledge, this study presents a distinctive methodology that integrates histopathological slide images with DL techniques to construct a predictive model for assessing the response to the first-line PD-1 inhibitor combined chemotherapy in advanced GC patients. Utilizing a cohort of 313 patient samples, we developed the ICIsNet and conducted a comprehensive retrospective testing encompassing both internal and external cohorts.

Recent trials have shown that for advanced GC, combining immunotherapy with chemotherapy is more effective than chemotherapy alone. The CheckMate 649 study [19], focusing on non-Asian patients, revealed that nivolumab with oxaliplatin-based chemotherapy improves response rates and survival times significantly compared to CAPOX or FOLFOX alone. Similarly, ATTRACTION-4 [34] showed the benefits of nivolumab with chemotherapy in Asian populations, without considering PD-L1 expression levels. These findings, supported by other studies like KEYNOTE-059, 061, and 062 [35], suggest that adding PD-1 inhibitors to chemotherapy could be a superior treatment approach for advanced-stage GC.

However, due to the high selectivity and expensive cost of ICIs, the choice of immunotherapy for patients requires careful consideration. Not all patients respond effectively to immunotherapy. Previous research has indicated that certain biomarkers such as PD-L1, tumor mutation burden (TMB), tumor-infiltrating lymphocytes (TILs), and microsatellite instability/defective mismatch repair (MSI/dMMR) can predict the population benefiting from immunotherapy. The reliability and clinical applicability of these biomarkers need further confirmation [36–38]. Therefore, the exploration of novel detection methods to more accurately predict the efficacy of ICIs is of great importance. Histopathological images contain numerous of information reflecting the cellular and molecular characteristics of tissues and can be transformed into quantitative data using image analysis software [39]. Thus, we can employ machine learning in a high-throughput manner to extract and quantify pathological image features for further assessment of the TME and tumor heterogeneity [40].

Digital pathology, involving the digitization of tissue slides and analysis through AI, has advanced cancer research, particularly with H&E-stained images [41]. AI’s initial role was to aid diagnoses [23], with studies like Mukhopadhyay et al. [42] showing digital WSI as reliable as traditional microscopy. Recent applications of DL on WSIs have provided detailed tumor assessments, achieving diagnostic accuracy on par with or surpassing pathologists [27, 43]. For instance, a study on breast cancer patients used DL to predict responses to chemotherapy from biopsy images, with high predictive accuracy (AUC of 0.847) [44]. Moreover, research by Armin Meier et al. [45] on gastric cancer employed DL to evaluate the tumor microenvironment’s impact on prognosis, yielding a CNN-derived risk score that outperformed traditional TNM staging in predictive value. These advancements underscore the potential of AI in enhancing the precision of cancer prognosis and treatment response predictions.

Building upon these precedents, we anticipated that AI could similarly extract subtle information from pre-treatment WSIs of advanced GC patients, thus enabling the development of predictive models for the response of PD-1 inhibitors combined chemotherapy. After training on tens of thousands of tiles extracted from WSI, our model exhibited impressive predictive capabilities in both our internal test cohort and multiple independent external test cohorts (with AUC values of 0.95, 0.92, and 0.96, 1 respectively). To our knowledge, this study represents the first instance to date of utilizing an AI model to extract information from WSIs and predict the efficacy of first-line PD-1 inhibitors combined chemotherapy in advanced GC.

Due to the complex architecture, black-box nature, and self-learning characteristics of neural networks, the selection of neural networks is often challenging to explain. In our study, to address this issue, we employed a strategy of generating heatmaps for the entire WSI based on the output scores of each tile. We aimed to interpret the neural network’s output using these heatmaps. Through visualization of heatmaps, we have identified significant features captured by our model. We observed that diffuse gastric cancer tissues, particularly signet ring cell carcinoma and mucinous adenocarcinoma, with fewer lymphocytic infiltrations tend to exhibit poor responses to immunotherapy. Conversely, regions with stronger cell adhesion, lower cellular and tissue heterogeneity, and more abundant lymphocytic infiltration demonstrate a more sensitive response to immunotherapy. Some studies have confirmed that poorly differentiated and highly diffuse GCs exhibit weak responses to immunotherapy. Jing Chen et al. utilized single-cell sequencing techniques to explore the Tumor Immune Microenvironment (TIME) of signet ring cell carcinoma. They discovered that compared to well-differentiated types, the TIME of this subtype appears inert, with both CD4 + and CD8 + T cells showing difficulty in mobilization, leading to poor or even no response to immunotherapy [46]. Additionally, Jie-Hai Yu and others investigated the response to PD-1 inhibitors in colorectal cancer patients and found that patients with signet ring cell carcinoma and mucinous adenocarcinoma exhibited weaker responses to immunotherapy, thereby indicating a higher risk of poor outcomes and prognosis [47]. The correlation between higher lymphocytic infiltration and better immunotherapy outcomes has been widely established. This relationship is significant because the activation of the PD-1/PD-L1 pathway necessitates the recruitment of a substantial number of immune cells [48]. Essentially, the presence of these immune cells within the tumor microenvironment plays a pivotal role in how effectively the immune system can identify and attack cancer cells under the modulation of immunotherapies targeting checkpoint inhibitors like PD-1/PD-L1. These dynamic underlines the critical importance of immune cell presence for the success of immunotherapeutic strategies [49]. Our study has demonstrated that DL models can capture essential features critical to the efficacy of immunotherapy. This not only provides clinicians with effective predictive tools but also sets the direction for future larger-scale and prospective research.

This study has several limitations that should be acknowledged. Firstly, the relatively small sample size could potentially impact the model’s ability to generalize due to tumor heterogeneity, although promising results were achieved across multiple independent external testing sets. Secondly, the complexity of pathological slides necessitated manual delineation of regions of interest, which could reduce practicality; our group is exploring semi-supervised learning methods to address this issue. Thirdly, variations in PD-1 inhibitors used and the need for longer follow-up periods due to pseudoprogression introduce additional challenges. We are currently expanding the sample size included in our training set, increasing the number of validating institutions, and designing prospective trials to verify the robustness of our model. Moving forward, it is hoped that these limitations will be addressed and the results of our study refined.

In summary, our study introduces the ICIsNet model, a DL-driven tool that utilizes ICIsRS based on histopathological images to forecast the sensitivity of advanced-stage GC to immunotherapy. This predictive capability can significantly inform clinical decisions, guiding clinicians in selecting patients most likely to benefit from immunotherapeutic approaches. In the future, ICIsNet may enhance treatment outcomes by personalizing therapeutic strategies, potentially reducing the exposure of non-responsive patients to the adverse effects of ineffective treatments in advanced-stage GC, which has the potential to exploit the way for personalized treatment strategies in advanced stage GC.

### Electronic supplementary material

Below is the link to the electronic supplementary material.

Supplementary Material 1

Supplementary Material 2

Supplementary Material 3

## Data Availability

Data is available under request.
