# Supplementary material for: Deep learning based digital pathology for predicting treatment response to first-line PD-1 blockade in advanced gastric cancer
Source: J Transl Med. 2024 May 8;22:438. doi: 10.1186/s12967-024-05262-z (PMC11077733; doi:10.1186/s12967-024-05262-z)
Supplement: Supplementary file 3 — Supplementary Material 3 [file 12967_2024_5262_MOESM3_ESM.docx]

| **Supplementary Table 1. Comparison of Models' Metrics and Selection on the FAH-NAU Dataset.** | | | | | | | |
| --- | --- | --- | --- | --- | --- | --- | --- |
|  | **ACC** | **AUC** | **PPV** | **NPV** | **SENS** | **SPEC** | **F1 score** |
| **DenseNet121** | 0.775 | 0.830 | 0.775 | 0.698 | 0.906 | 0.484 | 0.847 |
| **EfficientNet-B4** | 0.704 | 0.803 | 0.890 | 0.515 | 0.650 | 0.822 | 0.752 |
| **Swin Transformer-V2** | 0.768 | 0.817 | 0.817 | 0.640 | 0.853 | 0.577 | 0.835 |
| **Ensemble Model** | 0.768 | 0.824 | 0.837 | 0.630 | 0.817 | 0.663 | 0.827 |
| ACC: accuracy; AUC: area under the curve; PPV: positive predictive value; NPV: negative prediction value; SENS: sensitivity; SPEC: specificity | | | | | | | |

| **Supplementary Table 2. Comparison of Models' Metrics and Selection on the SAH-SYSU Dataset.** | | | | | | | |
| --- | --- | --- | --- | --- | --- | --- | --- |
|  | **ACC** | **AUC** | **PPV** | **NPV** | **SENS** | **SPEC** | **F1 score** |
| **DenseNet121** | 0.687 | 0.838 | 0.620 | 0.871 | 0.938 | 0.458 | 0.743 |
| **EfficientNet-B4** | 0.768 | 0.843 | 0.756 | 0.779 | 0.772 | 0.764 | 0.764 |
| **Swin Transformer-V2** | 0.753 | 0.824 | 0.720 | 0.795 | 0.810 | 0.700 | 0.762 |
| **Ensemble Model** | 0.770 | 0.856 | 0.722 | 0.837 | 0.859 | 0.686 | 0.785 |
| ACC: accuracy; AUC: area under the curve; PPV: positive predictive value; NPV: negative prediction value; SENS: sensitivity; SPEC: specificity | | | | | | | |

| **Supplementary Table 3. Comparison of Models' Metrics and Selection on the ACH-GZMU Dataset.** | | | | | | | |
| --- | --- | --- | --- | --- | --- | --- | --- |
|  | **ACC** | **AUC** | **PPV** | **NPV** | **SENS** | **SPEC** | **F1 score** |
| **DenseNet121** | 0.700 | 0.826 | 0.447 | 0.902 | 0.792 | 0.662 | 0.571 |
| **EfficientNet-B4** | 0.845 | 0.887 | 0.702 | 0.892 | 0.685 | 0.900 | 0.693 |
| **Swin Transformer-V2** | 0.686 | 0.908 | 0.445 | 0.958 | 0.922 | 0.604 | 0.601 |
| **Ensemble Model** | 0.824 | 0.904 | 0.618 | 0.929 | 0.815 | 0.826 | 0.703 |
| ACC: accuracy; AUC: area under the curve; PPV: positive predictive value; NPV: negative prediction value; SENS: sensitivity; SPEC: specificity | | | | | | | |
