# Supplementary material for: Deep learning based digital pathology for predicting treatment response to first-line PD-1 blockade in advanced gastric cancer
Source: J Transl Med. 2024 May 8;22:438. doi: 10.1186/s12967-024-05262-z (PMC11077733; doi:10.1186/s12967-024-05262-z)
Supplement: Supplementary file 1 — Supplementary Material 1 [file 12967_2024_5262_MOESM1_ESM.docx]

Dear Editor,

First and foremost, we would like to express our heartfelt gratitude for the careful assessment and constructive comments provided by you and the peer reviewers regarding our manuscript. The insights and expertise offered by the reviewers were incredibly invaluable. We have addressed each point in a detailed response and have made comprehensive amendments to our manuscript to address the concerns raised.

In response to the reviewers' concerns about sample size, we have expanded our cohort significantly, incorporating an additional 78 patients from multiple centers, which represents an increase of over 40% to the original dataset. The manuscript and its accompanying materials, including updated tables and figures, have been revised accordingly to reflect these changes.

We are pleased to resubmit our revised manuscript titled "Deep Learning based Digital Pathology for Predicting Treatment Response to First-Line PD-1 Blockade in Advanced Gastric Cancer" to the Journal of Translational Medicine. With the enlarged training and validation cohorts, we believe our study now exhibits stronger generalizability. Our research provides a precise and personalized approach to predict the response of patients with advanced gastric cancer to PD-1 blockade combined with chemotherapy. Moreover, it lays the groundwork for future research into the applications of artificial intelligence in this domain. We believe our findings make a substantial contribution to the current understanding and management of gastric cancer, aligning with the high-impact nature of your esteemed journal.

Thank you for considering our resubmission.

Sincerely,

Guanghua Li
