# Supplementary material for: Deep learning based digital pathology for predicting treatment response to first-line PD-1 blockade in advanced gastric cancer
Source: J Transl Med. 2024 May 8;22:438. doi: 10.1186/s12967-024-05262-z (PMC11077733; doi:10.1186/s12967-024-05262-z)
Supplement: Supplementary file 2 — Supplementary Material 2 [file 12967_2024_5262_MOESM2_ESM.docx]

**Point-by-point response to Comments and Suggestions for Authors**

**Reviewer1:**

**Comments 1:** The title should clearly reflect the focus on utilizing deep learning for predicting treatment response in advanced gastric cancer patients.

**Response:** We deeply appreciate your insightful comments, acknowledging the vital importance of an appropriate title for our study. After thoroughly consideration of your suggestion, we have revised the title to "Deep Learning-based Digital Pathology for Predicting Treatment Response to First-Line PD-1 Blockade in Advanced Gastric Cancer." (***please see lines 2-3 in the revised manuscript***) We believe this new title more accurately reflects the essence and findings of our research, emphasizing the innovative approach and its clinical implications.

**Comments 2:** The background should further elaborate on the advantages and necessity of deep learning in this field of study.

**Response:** Thank you for your valuable comments. It’s important to emphasize on the application of deep learning in the field of oncology, particularly in the context of treatment response. Accordingly, we have enriched the background section with content that highlights this application, emphasizing the necessity and relevance of deep learning techniques in the field (***please see lines 62-64 in the revised manuscript***). We believe these additions will substantially enhance the readers’ understanding of the study's context and its implications.

**Comments 3:** The results section is concise and informative. It could be enhanced by briefly explaining the importance of AUC values in assessing model performance, to make it accessible to a broader audience. Mentioning the exact AUC values rather than a range might provide a clearer understanding of the model's performance.

**Response:** Thank you for your insightful suggestion. After careful consideration, we have revised the abstract's results section and the corresponding section in the main manuscript to reflect specific AUC values rather than a range (***please see lines 76-79 and 429-431 in the revised manuscript***). Additionally, we have included a description of the importance of AUC values in the assessment of model performance (***please see lines 433-437 in the revised manuscript***). These amendments aim to provide clarity and enhance the reader's understanding of our model's predictive accuracy and its significance within the study.

**Comments 4:** The conclusion effectively summarizes the study's significance. Consider emphasizing the potential clinical implications of your findings, such as how ICIsRS could influence decision-making in treating advanced GC patients.

**Response:** We are grateful for your insightful suggestion. The conclusion is indeed crucial as it illustrates the novel contribution of our work to the existing field. Therefore, we have incorporated additional emphasis on the potential impact of our study's findings on current clinical decision-making processes (***please see lines 82-83 and 595-601 in the revised manuscript***). This enhancement highlights the practical implications and the new value our research offers to the domain.

**Reviewer2:**

**Comments 1:** Please pay attention to the quality of the manuscript. Ensure the consistency and readability of all the Table data. Also, pay attention to the uniformity of all significant figures in the paper.

**Response:** Thank you for your insightful suggestions. We apologize for the oversight regarding the consistency and visualization of tables and figures in our manuscript. In response, we have thoroughly reviewed and proofread all tables and figures and have redrawn the majority of them (***please see Figure 1-4 and table 1-3 in the revised manuscript***). Additionally, we have incorporated more visual aids, such as confusion matrices, to enhance the presentation of our data. We believe these efforts will significantly improve the quality of our article and are hopeful for your positive feedback.

**Comments 2:** The reviewer noticed that the ages of all patients in Table 1 are concentrated between 55 and 59 years old. Is this due to the limitations of the sample itself? Or does it indicate that the patient's age of illness is usually within this age range?

**Response:** Thank you for your observation regarding the age distribution presented in Table 1 of our manuscript. Indeed, the baseline characteristics show that the average and median ages of patients across the four cohorts fall within the 55–65-year age bracket. However, the age range for each cohort spans over 40 years, ***as indicated by the minimum and maximum values in Table 1***. This distribution aligns with epidemiological findings in Asian populations, where the incidence of gastric cancer begins to increase significantly after the age of 50 and peaks around the age of 70^1, 2^. Additionally, there is a trend towards younger ages of onset in recent years. Our cohort's age distribution is consistent with these characteristics. The study adheres to strict inclusion and exclusion criteria without any special exceptions beyond these standards.

**Comments 3:** Analyzing 186 patient samples is indeed a relatively small sample size, and this could potentially impact the model. Tumor heterogeneity is a significant factor in cancer, and with a smaller sample size, there is a risk that the model may not capture the full spectrum of heterogeneity present in advanced GC. As a result, there may be limitations in the model's ability to accurately predict responses in a broader patient population. The reviewer suggests that to address the concern about sample size and potential limitations in generalization, future studies could consider expanding the dataset to include a larger and more diverse cohort of patients. This would help validate the model's performance across a broader range of tumor heterogeneity and clinical scenarios. Additionally, ongoing validation studies with larger patient cohorts could further confirm the model's predictive accuracy and reliability in real-world clinical settings.

**Response:** Thank you for your perceptive observations and valuable suggestions concerning our sample size and the potential impact on the model's generalizability. We acknowledge that the limited sample size, necessitated by the recent integration of PD-1 combined chemotherapy in CSCO guidelines in 2021 and FDA approval in 2022, poses constraints, especially considering our follow-up cut-off in May 2023.

Nevertheless, we endeavored to address this limitation. During the manuscript revision period, we extended follow-up and continued enrollment from May 2023 to January 2024, in strict adherence to our inclusion and exclusion criteria. ***This enabled us to augment the FAH cohort by 47 patients, the FAH-NCU cohort by 29, and the GZMU cohort by 2 (please see revised Table 1).*** These new additions allowed us to enrich our dataset and bolster the robustness of our findings. Specifically, the 47 additional patients from the FAH cohort were randomized into training and internal testing sets at an 8:2 ratio, while the rest were incorporated into their respective external testing cohorts. The model was retrained, validated, tested, and visualized with this expanded data. ***Ultimately, our total cohort grew to 264 patients with 313 WSIs, with testing results maintaining all AUC values above 0.9 (please see revised Table 3, Figure 3)***. We hope this expanded cohort may address the potential limitations in the model's generalizability due to the previously limited sample size.

All the other changes related to the expansion of the sample size are clearly marked in the revised manuscript. Additionally, we further refined the results and discussion sections to provide more detailed insights into the interpretability and test results of the model (***please see lines 451-457, 539-570***).

Despite our efforts to expand the sample size, we still face the limitation of relatively limited samples. Therefore, we further elaborate on these constraints and the need for future large-scale, prospective research (***please see lines 578-582)***. This acknowledgment helps set the stage for further studies and emphasizes the need for broader collaboration and resource allocation to overcome these challenges and enhance the validity of our findings.

**Comments 4:** The author should provide scale bars in Figure4 for the reader's easy comparison and observation.

**Response:** Thank you for your insightful suggestion regarding the inclusion of scale bars in Figure 4. We agree that scale bars indeed facilitate the reader’s understanding and ease of comparison. Accordingly, ***we have added scale bars to Figure 4 in the revised manuscript***. This addition should enhance the clarity and interpretability of the visual data presented.

**Comments 5:** References only include a few recently published papers. The reviewer suggests incorporating more recently published articles to strengthen the literature review and ensure the most up-to-date information is included.

**Response:** Thank you for your valuable suggestion to update our literature references. ***We have reviewed and revised our citations, replacing all outdated references with the most recent literature to ensure that our manuscript reflects current research and insights***. Your meticulous attention to detail is greatly appreciated and has contributed significantly to the enhancement of our study.

1.	Li Y, Ren N, Zhang B, et al. Gastric cancer incidence trends in China and Japan from 1990 to 2019: Disentangling age-period-cohort patterns. Cancer. 2023;129(1).

2.	Smyth EC, Nilsson M, Grabsch HI, et al. Gastric cancer. Lancet. 2020;396(10251):635-648.
